# RPL19 Is a Prognostic Biomarker and Promotes Tumor Progression in Hepatocellular Carcinoma

**DOI:** 10.3389/fcell.2021.686547

**Published:** 2021-07-19

**Authors:** Benchen Rao, Jianhao Li, Tong Ren, Jing Yang, Guizhen Zhang, Liwen Liu, Haiyu Wang, Maoxin Huang, Zhigang Ren, Zujiang Yu

**Affiliations:** ^1^Department of Infectious Diseases, The First Affiliated Hospital of Zhengzhou University, Zhengzhou, China; ^2^Precision Medicine Center, Gene Hospital of Henan Province, The First Affiliated Hospital of Zhengzhou University, Zhengzhou, China; ^3^Department of Breast Surgery, The Affiliated Cancer Hospital of Zhengzhou University, Zhengzhou, China; ^4^Department of Pharmacy, The First Affiliated Hospital of Zhengzhou University, Zhengzhou, China; ^5^Department of Dermatology, The First Affiliated Hospital of Zhengzhou University, Zhengzhou, China

**Keywords:** hepatocellular carcinoma, ribosomal protein L19, weighted gene co-expression network analysis, prognostic biomarker, immune infiltration

## Abstract

**Background:**

Hepatocellular carcinoma (HCC) is one of the most common malignancies, and the therapeutic outcome remains undesirable due to its recurrence and metastasis. Gene dysregulation plays a pivotal role in the occurrence and progression of cancer, and the molecular mechanisms are largely unknown.

**Methods:**

The differentially expressed genes of HCC screened from the GSE39791 dataset were used to conduct weighted gene co-expression network analysis. The selected hub genes were validated in The Cancer Genome Atlas (TCGA) database and 11 HCC datasets from the Gene Expression Omnibus (GEO) database. Then, a tissue microarray comprising 90 HCC specimens and 90 adjacent normal specimens was used to validate the hub genes. Moreover, the Hallmark, Gene Ontology (GO) and Kyoto Encyclopedia of Genes and Genomes (KEGG) databases were used to identify enriched pathways. Then, we conducted the immune infiltration analysis.

**Results:**

A total of 17 co-expression modules were obtained by weighted gene co-expression network analysis. The green, blue, and purple modules were the most relevant to HCC samples. Four hub genes, RPL19, RPL35A, RPL27A, and RPS12, were identified. Interestingly, we found that all four genes were highly expressed in HCC and that their high expression was related to a poor prognosis by analyzing the TCGA and GEO databases. Furthermore, we investigated RPL19 in HCC tissue microarrays and demonstrated that RPL19 was overexpressed in tumor tissues compared with non-tumor tissues (*p* = 0.016). Moreover, overexpression of RPL19 predicted a poor prognosis in hepatocellular carcinoma (*p* < 0.0007). Then, enrichment analysis revealed that cell cycle pathways were significantly enriched, and bile acid metabolism-related pathways were significantly down-regulated when RPL19 was highly expressed. Furthermore, immune infiltration analysis showed that immune response was suppressed.

**Conclusion:**

Our study demonstrates that RPL19 may play an important role in promoting tumor progression and is correlated with a poor prognosis in HCC. RPL19 may serve as a promising biomarker and therapeutic target for the precise diagnosis and treatment of HCC in the future.

## Introduction

Liver cancer is one of the leading causes of global disease burden worldwide, with 42,810 new cases and 30,160 deaths in 2020 (Yu and Schwabe, 2017; Siegel et al., 2020). Hepatocellular carcinoma (HCC) is the most frequent and common type of primary liver cancer and is attributed mainly to the progression of chronic liver disease. Most HCC patients are diagnosed in the advanced stage, and it has been reported that the 5-year recurrence rate is more than 70% (Rahbari et al., 2011). In view of the high incidence and mortality of HCC, it is imperative to find a novel biomarker for diagnosis, prognosis and treatment to improve the patient survival rate.

With the development of high-throughput research methods, precious resources for the analysis of whole-genome co-expression networks and screening of tumor biomarkers associated with prognosis and phenotypes have been provided by a large public transcriptome database. Weighted gene co-expression network analysis (WGCNA) is suitable for multisample complex data analysis and can be used to analyze the relationship between gene clusters and sample phenotypes and the networks between genes in gene sets to identify key transition genes (Panwar et al., 2021). Currently, systematic biological analysis has been widely used to identify diagnostic and prognostic markers and therapeutic targets. For instance, two modules and 10 hub genes identified by Zhang et al. (2018) were related to the tumorigenesis of oral squamous cell carcinoma. Two cervical squamous cell carcinoma-related hub modules and 116 hub genes were identified by the WGCNA method (Liu et al., 2019). Ribosomal protein L19 (RPL19) as a hub gene was identified in HCC by WGCNA in this study. RPL19 is a member of the ribosomal protein family that assembles to form small and large ribosomal subunits. RPL19 has been reported as a biomarker for many cancers (Dressman et al., 2003; Bee et al., 2006; Huang et al., 2008). However, the diagnostic, prognostic and therapeutic value of RPL19 in HCC has not been investigated.

In this study, WGCNA was conducted based on the GSE39791 dataset, which included 144 HCC and paracancerous tissues. After screening, 54 pairs of HCC and paracancerous tissues were selected to identify 17 co-expression modules and four hub genes (RPS12, RPL19, RPL35A, and RPL27A). Then, we screened the above four genes again in The Cancer Genome Atlas (TCGA) and Gene Expression Omnibus (GEO) databases. Subsequently, we validated RPL19 in an HCC tissue microarray. RPL19 was speculated to be a prognostic biomarker and promote tumor progression in HCC.

## Materials and Methods

### Datasets

**FIGURE 1:**
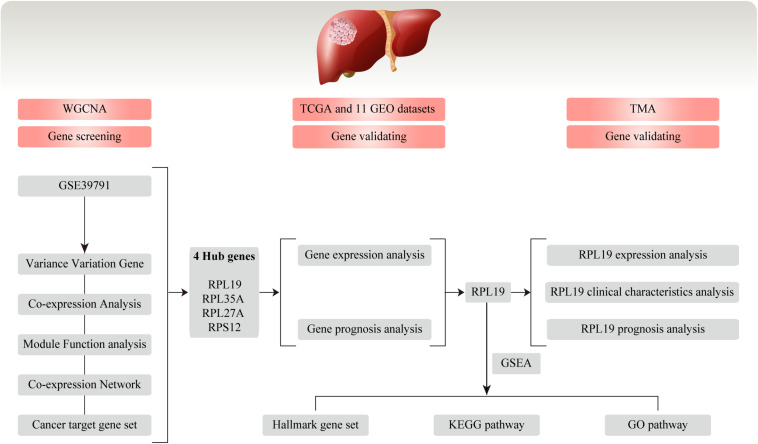
Flow chart for this study. In this study, we conducted WGCNA and screened four hub genes (RPL19, RPL35A, RPL27A, and RPS12) based on the GEO database GSE39791. Then, the four hub genes were validated in TCGA database and 11 GEO datasets. Moreover, we chose RPL19 as a key gene and validated it in the TMA. WGCNA, weighted gene co-expression network analysis; GEO, Gene Expression Omnibus; TCGA, The Cancer Genome Atlas; TMA, tissue microarray; HCC, hepatocellular carcinoma; GSEA, gene set enrichment analysis; KEGG, Kyoto Encyclopedia of Genes and Genomes; GO, Gene Ontology.

The study design is shown in a flow diagram (Figure 1). GSE39791 tissue chip data for 144 cancer samples (72 pairs of HCC and paracancerous samples paired one by one) were downloaded from the GEO database^1^, and corresponding sample information was used to conduct WGCNA (Kim et al., 2014). Then, to verify the results of the above analysis, we searched the GEO and TCGA database again. A total of 369 liver cancer data and 50 non-tumor data points were obtained from the TCGA database^2^. We used the GEO to gather and analyze 11 liver cancer mRNA microarray datasets. BRB-array tools were used to determine the differentially expressed genes between HCC tissues and normal liver tissues in each dataset. Detailed information is shown in Table 1. The human protein–protein interactions (PPI) were compiled from the Human Integrated Protein–Protein Interaction rEference (HIPPIE) database^3^ (Misselbeck et al., 2019).

**TABLE 1:** HCC expression profile cohorts used in this study.

Cohort ID	Platforms	HCC tissue	Paracancerous tissue	Year	Country
TCGA	Illumina	369	50	2009	United States
GSE14520	Affymetrix	225	220	2010	United States
GSE39791	Illumina	72	72	2014	United States
GSE76297	Affymetrix	153	151	2017	United States
GSE54236	Agilent	81	80	2014	Italy
GSE62232	Affymetrix	81	10	2014	France
E	Affymetrix	95	39	2014	Taiwan
GSE60502	Affymetrix	18	18	2015	Taiwan
GSE57957	Illumina	39	39	2014	Singapore
GSE76427	Illumina	115	52	2017	Singapore
e	Affymetrix	60	65	2016	Switzerland
GSE102083	Affymetrix	156	105	2018	Japan
Total		1464	901		

*HCC, hepatocellular carcinoma; TCGA, The Cancer Genome Atlas.*

### Construction of the Co-expression Network

Weighted gene co-expression network analysis is a systematic biology method that uses gene expression data to construct a scale-free network. WGCNA analyzes thousands of genes with the greatest changes instead of genes that are differentially expressed, and at the same time it converts the associations between thousands of genes and phenotypes into associations between several gene sets and phenotypes, eliminating the problem of multiple hypothesis testing and correction. First, we selected the expression data of genes that changed in each sample (seed genes) and used the R software package WGCNA to construct a weighted gene co-expression network. We calculated the coefficient of variation (CV) for each gene and chose 3.6 as the cut-off value to identify the differentially expressed genes (DEGs). Then, a soft threshold of β = 6 was chosen to ensure that the co-expression network was a scale-free distribution. And we screened the co-expression module. Next, the expression matrix was converted to an adjacency matrix and then to a topological matrix. Based on TOM, clustering was accomplished by using the average linkage algorithm. In accordance with the dynamic hybrid tree cutting algorithm, the minimum number of genes (lncRNAs) in the network module was set to 30. We calculated the eigengenes of each module, conducted cluster analysis on the modules, merged the close modules into new modules and set the height = 0.25. The higher the correlation coefficient is, the more important the module. According to the expression relationship of the genes in each co-expression module, we chose the co-expression pairs whose co-expression weights were larger than 0.1 as the edges of the final co-expression network.

### Tissue Samples

The tissue microarray (TMA) from Asians containing 90 normal liver specimens and 90 HCC specimens (HLiv-HCC180Sur-15) from cancer-adjacent tissues was purchased from Shanghai Outdo Biotech Co., Ltd. We further validated RPL19 expression and its prognostic value in HCC by TMA. None prior radiotherapy, immunotherapy or chemotherapy were conducted on the patients whose samples were included in the TMA before surgery. This study was approved by the Ethics Committee of The First Affiliated Hospital of Zhengzhou University, Zhengzhou, China.

### Immunohistochemistry (IHC) Staining

Immunohistochemistry staining was carried out as described previously (Cui et al., 2019). According to the Remmele scoring system (Remmele et al., 1986), four fields of view were randomly selected under low and high power, 100 cells were counted in each field, and the percentage of RPL19 cytoplasmic staining in each field of power was calculated as a percentage of positive cells. Two experienced pathologists separately evaluated the immunostained samples. The results were divided into four groups: score 1, <25%; score 2, 25%∼50%; score 3, 50%∼75%; and score 4, >75%. Scores of 1 and 2 were defined as low expression, and scores of 3 and 4 were defined as high expression.

### Biological Functional Analysis

The Metascape software^4^ was used to analyze the functional gene clustering. The Kyoto Encyclopedia of Genes and Genomes (KEGG) database^5^, Gene Ontology (GO) gene sets^6^ and Hallmark gene sets^7^ were used to conduct gene set enrichment analysis (GSEA).

### Single-Sample Gene Set Enrichment Analysis (ssGSEA)

The ssGSEA in R package gsva was used to quantify the infiltration levels of the immune cell types. SsGSEA applies gene signatures expressed by immune cell populations to individual cancer samples. We used the deconvolution approach to analyze the immune cells involved innate immunity and adaptive immunity.

### Statistical Analysis

All statistical tests and graphing were performed using R software (version 3.4.3)^8^ and GraphPad Prism 7.0 (GraphPad Software, San Diego, United States). Differences between two groups were analyzed by Student’s *t*-test. Clinicopathologic variables were analyzed by chi-square tests. The overall survival (OS), relapse-free survival (RFS), and progress-free survival (PFS) of HCC patients were calculated with Kaplan–Meier curves and log-rank tests. GSEA was used to determine which gene sets were associated with the expression of hub genes in datasets. *P* < 0.05 was considered to be statistically significant.

## Results

### Construction of the Weighted Co-expression Network

**FIGURE 2:**
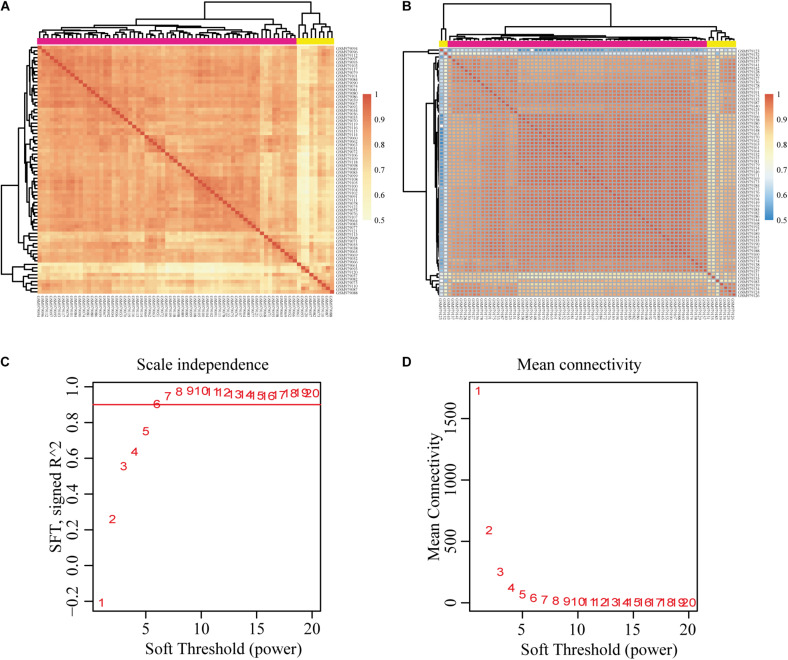
Correlation analysis of each sample and construction of the weighted gene co-expression network based on GEO database GSE39791. **(A)** Correlation between HCC samples. **(B)** Correlation between paracancerous samples. **(C,D)** Analysis of network topology for various soft-thresholding powers. Pearson’s correlation test was used to assess the correlation between samples. HCC, hepatocellular carcinoma; GEO, Gene Expression Omnibus.

We downloaded the raw data of GSE39791, which includes 144 HCC and paracancerous tissues, to construct the gene co-expression networks. Results of the cluster analysis of the correlation between samples are shown in Figure 2. However, it can be clearly seen that the correlations between HCC samples can be divided into two groups (Figure 2A), and the intragroup correlation was high, which showed that these samples had some heterogeneity. The paracancerous tissue samples could be divided into three groups because two samples had weak correlations (Figure 2B). We chose the group with the highest correlation as the datasets for this study. Given that the cancer and paracancerous samples were paired, we ultimately selected 54 pairs of samples. A total of 31,334 genes were obtained (Supplementary Table 1). Then, 7,814 DEGs were identified (Supplementary Table 2).

**FIGURE 3:**
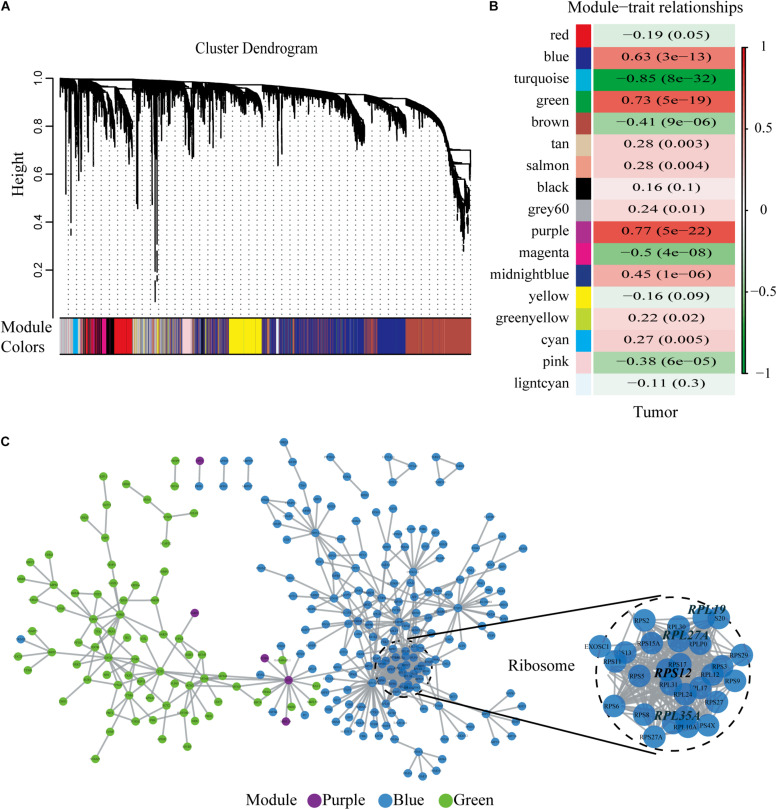
Selected hub modules and the human PPI network. **(A)** Gene dendrogram and module colors. **(B)** Relationship between modules and traits. The figure showed the correlation coefficients and the *P*-values. Red meant positive correlation, while green meant negative correlation. **(C)** Human PPI network was conducted based on the three hub modules. PPI, protein–protein interaction.

We chose β = 6 to ensure that the co-expression network was scale free (Figures 2C,D). Then, a total of 17 modules were obtained (Figure 3A). The gene statistics in each module are shown in Supplementary Table 3. Overall, 7814 genes were allocated into 17 modules (Supplementary Table 4), and the gray module included all the genes that could not be clustered. The Pearson correlation coefficients between each module eigengene (ME) and sample trait were calculated (Figure 3B). We can conclude that these three modules (green, blue and purple) are the most relevant to HCC samples. In addition, we used the R package clusterProfiler to conduct the KEGG enrichment analysis and GO enrichment analysis of 17 modules. The results showed that 11 modules were significantly enriched in 121 KEGG pathways (Supplementary Table 5 and Supplementary Figure 1). The green module was enriched in 6 KEGG pathways, including 2 cancer-related pathways (DNA replication and the cell cycle). Additionally, the blue module was enriched in ribosomes, RNA transport and necroptosis, which are closely related to tumorigenesis and progression. These results imply that both the green and blue modules are closely related to tumorigenesis and progression. The co-expression network contained a total of three gene modules, and their distribution is shown in Supplementary Figure 2 and Supplementary Tables 6, 7. As the gene node degree increased, the number of nodes decreased.

### Identification of the Four Hub Genes by the Human Protein–Protein Interaction Network

The human PPI network contained 17,381 nodes with 19.6 neighboring nodes on average (Supplementary Table 8). Then, all the responsive genes were mapped to the human PPI network. In total, 1148 genes were covered in the PPI network. Among them, 265 interacted with and co-expressed 2.33 neighboring nodes on average (Figure 3C and Supplementary Table 9). Through topological property and biological enrichment analyses, it was finally determined that the screening threshold was at least 10% of co-expressed genes in the neighboring nodes, and genes whose FDR-corrected *p*-value was less than 0.05 were defined as significantly differentially expressed in the co-expression-interaction gene enrichment analysis. The results are shown in Table 2. As shown in the table, four genes, RPL19, RPS12, RPL27A, and RPL35A, were obtained. These four genes are related to ribosomes. According to the literature, RPS12 is a tumor marker for liver cancer (Wang et al., 2009).

**TABLE 2:** Screened cancer-specific genes.

Gene symbol	Number of co-expression neighborhood gene	Number of neighborhood gene	Number of co-expression gene	Number of network gene	Co-expression neighborhood gene per	Fisher’s exact test *p*-value	FDR
RPL19	34	158	1148	17381	0.177083	1.18E−07	0.000137
RPS12	26	115	1148	17381	0.184397	1.61E−06	0.00185
RPL27A	29	145	1148	17381	0.166667	3.51E−06	0.004005
RPL35A	20	86	1148	17381	0.188679	1.76E−05	0.01978

*FDR, false discovery rate.*

### Expression, Clinicopathological, and Prognostic Analyses of the Four Hub Genes in the GEO and TCGA Databases

**FIGURE 4:**
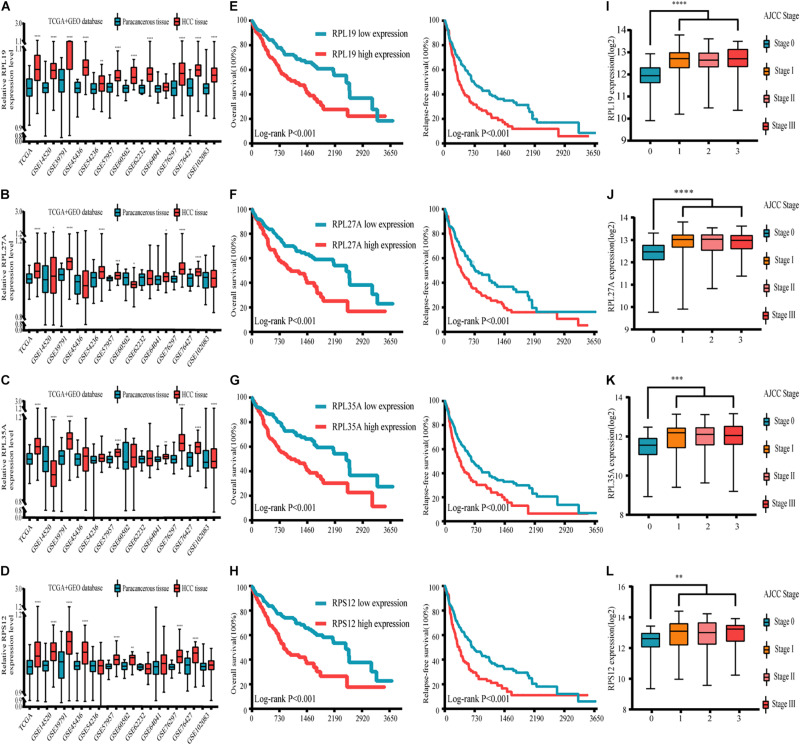
Expression, clinicopathological and prognostic analyses of the four hub genes in the GEO and TCGA databases. **(A–D)** Expression of the four hub genes in the GEO and TCGA databases. **(E–H)** Relationship between the expression levels of the four hub genes and the OS and RFS of HCC patients. **(I–L)** Expression levels of the four hub genes in different AJCC stages of HCC. TCGA database and 11 GEO datasets (including GSE14520, GSE39791, GSE45436, GSE54236, GSE57957, GSE60502, GSE62232, GSE64041, GSE76297, GSE76427, and GSE102083) were used for analysis in this section. Differences between two groups were analyzed by Student’s *t*-test. The OS and RFS of HCC patients were calculated with Kaplan–Meier curves. GEO, Gene Expression Omnibus; TCGA, The Cancer Genome Atlas; OS, overall survival; RFS, relapse-free survival; HCC, hepatocellular carcinoma; GSEA, gene set enrichment analysis. *P < 0.05, **P < 0.01, ***P < 0.001, ****P < 0.0001.

Through the analysis and comparison of 11 HCC datasets in the GEO database, we found that the expression levels of RPL19 were significantly higher in HCC tissues than in paracancerous tissues in 10 datasets (*P* < 0.05). Moreover, RPL27A was markedly increased in eight datasets, and RPL35A and RPS12 were markedly increased in eight datasets (Figures 4A–D). In addition, we compared 369 liver cancer samples and 50 paracancerous samples in the TCGA database. The results showed that the expression levels of the four hub genes were significantly higher in HCC tissues than in paracancerous tissues (*P* < 0.0001). The expression levels of the four hub genes in different stages of liver disease are shown in Supplementary Figure 3. In addition, the relationship between different expression levels of RPL19, RPL27A, RPL35A, and RPS12 and the clinical prognosis of patients was compared through survival analysis. The OS and relapse-free survival (RFS) of HCC patients with high expression of the four hub genes were significantly shorter than those with low expression (Figures 4E–H). Among the HCC patients with TNM stage I∼II disease, OS and RFS were significantly shorter in those with high expression of the four hub genes than in those with low expression (*P* < 0.0001), and the same results were obtained from HCC patients with TNM stage III∼IV disease (*P* < 0.0001) (Supplementary Figure 4). Then, we investigated the expression levels of the four hub genes in different AJCC stages of HCC. The expression levels of the four hub genes were significantly higher in stages I, II, and III than in stage 0 (*P* < 0.01) (Figures 4I–L). The expression changes in these four genes in stages I, II, and III were not significantly different (*P* > 0.05), indicating that these four genes can be used as important molecular biomarkers for the early diagnosis of HCC. These four genes can also be used as prognostic biomarkers according to the results of the subsistence analysis.

From the above results, at the expression level of the four genes, we found that RPL19 has the most significant difference in the gene expression level between HCC and paracancerous tissues. Compared with the other three genes, the expression level of RPL19 was significantly different in the most GEO data sets. Moreover, the difference in the expression level of RPL19 between HCC and paracancerous tissues has the largest fold change value in the TCGA database. Therefore, judging from the expression levels of the four genes, we thought it was more meaningful to choose RPL19 for further research. Furthermore, at the protein level of the four molecules, we searched the Human Protein Atlas database and found that RPL19 was highly expressed in HCC tissues based on the staining intensity. However, the other three molecules were moderately expressed, lowly expressed or not expressed in HCC tissues. Therefore, judging from the protein level of the four molecules, we thought it was more meaningful to choose RPL19 for further research. At last, we focused on the functions of the four genes. We found that RPL19 served as a biomarker and was involved in the progression of multiple tumors except HCC. In summary, we comprehensively considered the expression level, protein level, and gene function of the four genes. Finally, we chose RPL19 for further research in HCC.

### RPL19 Is Closely Related to the Tumor Progress and the Poor Prognosis of HCC

**FIGURE 5:**
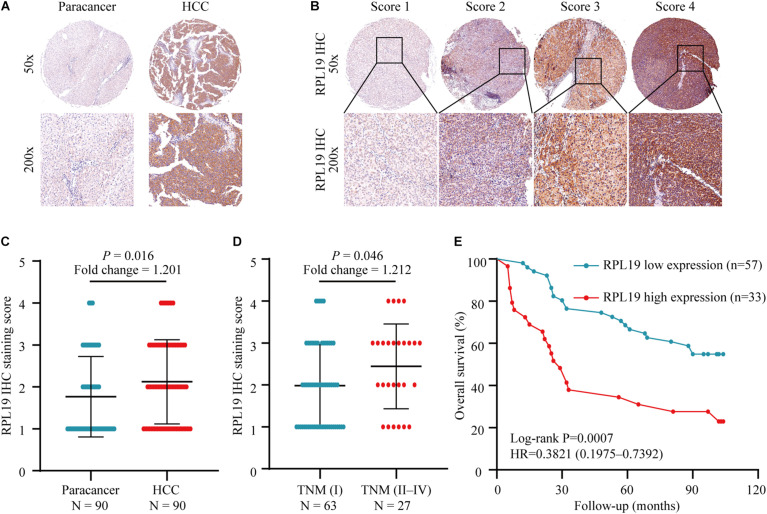
Relationship between the expression of RPL19 in hepatocellular carcinoma and clinical prognosis. **(A)** RPL19 expression was higher in HCC patient tumor tissues than in paired paracancerous tissues. **(B)** IHC analysis of RPL19 expression in the TMA. **(C)** Expression of the RPL19 protein in the TMA in HCC and paracancerous tissues. **(D)** Expression of the RPL19 protein in the TMA in different TNM stages. **(E)** Relationship between the expression of the RPL19 protein and patient OS in the TMA. Differences between two groups were analyzed by Student’s *t*-test. The OS of HCC patients were calculated with Kaplan–Meier curves. TMA, tissue microarray; IHC, immunohistochemistry; HCC, hepatocellular carcinoma; OS, overall survival.

The above results indicated that the four hub genes (RPL19, RPL27A, RPL35A, and RPS12) are closely related to the clinical prognosis of HCC. Through preliminary experiments and a literature search, we ultimately chose RPL19 for further research. According to the IHC staining intensity, the expression of the RPL19 protein in tumor tissues was significantly higher than that in paracancerous tissues (*P* = 0.016) (Figures 5A–C). The different levels of staining intensity are shown in Figure 5B. Furthermore, RPL19 expression was significantly positively related to alpha fetoprotein (AFP) and potentially positively related to the TNM stage in patients (Table 3). Moreover, the expression level of the RPL19 protein increased significantly with progression from TNM stage I to III (*P* = 0.046) (Figure 5D). Kaplan-Meier analysis also showed that the OS of HCC patients with low RPL19 expression was significantly longer than that of patients with high RPL19 expression (*P* = 0.0007) (Figure 5E). Finally, univariate and multivariate analyses demonstrated that, in addition to AFP and TNM stage, RPL19 might also be an independent prognostic factor for HCC patients (Table 4). The above results once again suggest that the expression level of RPL19 is upregulated in HCC tissues and closely related to the clinical prognosis of patients with liver cancer.

**TABLE 3:** Relationship between RPL19 expression and the clinicopathological features of hepatocellular carcinoma patients.

Clinicopathological feature		No. of patients (%)	RPL19 expression level		*P-value*
				
			Low	High	
Age (years)	≤50	40	25	15	0.883
	>50	50	32	18	
Sex	Female	10	4	6	0.104
	Male	80	53	27	
TNM	I	63	44	19	0.050*
	II-IV	27	13	14	
Tumor size	≤3 cm	36	23	13	0.929
	>3 cm	54	34	20	
AFP	≤300 μg/L	48	36	12	0.014*
	>300 μg/L	42	21	21	

*P < 0.05.TNM, tumor-node-metastasis; HR, hazard ratio; CI, confidential interval; RPL19, ribosomal protein L19; AFP, alpha fetoprotein.

**TABLE 4:** Univariate and multivariate analyses of the overall survival of hepatocellular carcinoma patients.

Clinicopathological feature	Univariate analysis			Multivariate analysis		
		
	HR	95% (CI)	*P-*value	HR	95% (CI)	*P-*value
Age (years)	1.018	0.987–1.049	0.255			
Sex	1.472	0.455–4.754	0.519			
TNM stage	1.707	1.039–2.807	0.035*			
Tumor size	1.082	0.982–1.193	0.112			
AFP	1.377	1.092–1.736	0.007**			
RPL19 expression	1.921	1.39–2.656	<0.001**	1.693	1.189–2.411	0.003**

*P < 0.05; **P < 0.01.TNM, tumor-node-metastasis; HR, hazard ratio; CI, confidential interval; RPL19, ribosomal protein L19; AFP, alpha fetoprotein.

### Functional Annotation and Immune Infiltration Analysis of RPL19

**FIGURE 6:**
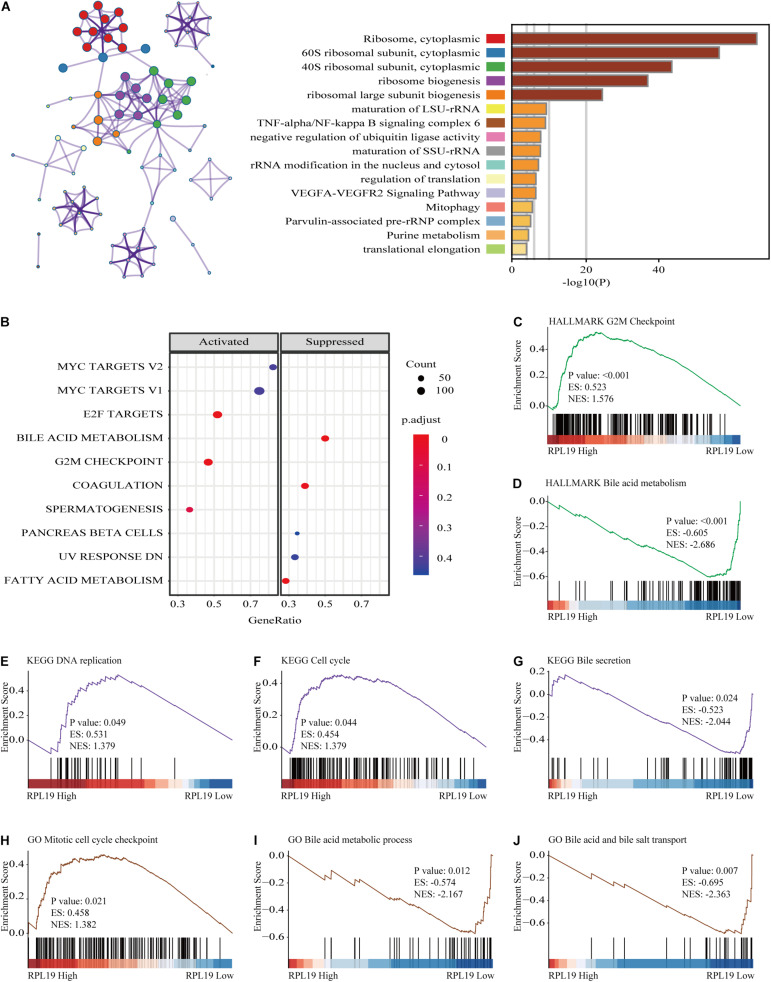
Functional annotation of RPL19. **(A)** Network and bar chart of 16 significantly enriched biological processes in HCC patients with high RPL19 expression. Each enriched node is presented in a different color. **(B)** The suppressed and activated hallmark pathways. **(C,D)** GSEA was conducted to determine the hallmark pathways. **(E–G)** GSEA was conducted to determine the KEGG pathways. **(H–J)** GSEA was conducted to determine the GO pathways. In this section, KEGG, HALLMARK, and GO database were used for analysis. GSEA, gene set enrichment analysis. KEGG, Kyoto Encyclopedia of Genes and Genomes; GO, Gene Ontology.

**FIGURE 7:**
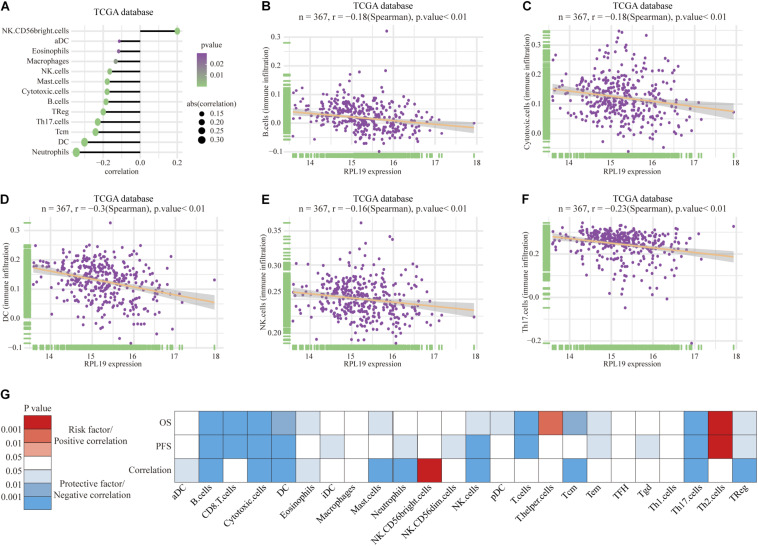
Immune infiltration analysis of RPL19. **(A–F)** Correlation between immune cells and the expression of RPL19, including B cells, Cytotoxic cells, DC, NK cells, and Th17 cells. **(G)** The heatmap of the correlation between OS, PFS and the expression of RPL19. In this section, TCGA database was used for analysis. Spearman’s correlation test was used to evaluate the correlation between the expression level of RPL19 and immune cells. DC, dendritic cells; NK cells, natural killer cells; OS, overall survival; PFS, progress free survival.

To further elucidate the mechanism by which RPL19 promotes the progression of HCC, we annotated the biological processes of RPL19 and conducted pathway analysis through Metascape. Metascape enrichment analysis revealed that “ribosome biogenesis,” “maturation of rRNA,” “TNF-alpha/NF-kappa B signaling pathway,” “negative regulation of ubiquitin ligase activity,” and “rRNA modification in the nucleus and cytosol” were enriched in biological processes and pathways that might be highly correlated with the malignant progression of HCC. Figure 6A showed the top 16 significantly enriched biological processes. Hallmark pathway analysis further revealed that “bile acid metabolism” and “fatty acid metabolism” were suppressed and that the “G2M checkpoint” was activated (Figure 6B). Then, gene set enrichment analysis (GSEA) was conducted to determine the hallmark pathways. The results indicated that cell cycle pathways were significantly enriched, and bile acid metabolism-related pathways were significantly downregulated when RPL19 was highly expressed (Figures 6C,D). In addition, GSEA was performed to investigate the enriched KEGG pathways. We found that “DNA replication” and “cell cycle” were significantly enriched and “bile secretion” was significantly downregulated (Figures 6E–G). Moreover, GSEA was performed to investigate the enriched GO pathways. The results showed that the mitotic cell cycle checkpoint pathway was enriched and that the bile acid transport and metabolic process pathway was downregulated (Figures 6H–J). The results showed similar pathways among the three enrichment analyses and indicate that RPL19 is associated with the tumorigenesis and progression of HCC. The immune infiltration analysis showed that activated dendritic cells (aDC), eosinophils, macrophages, natural killer cells (NK cells), mast cells, cytotoxic cells, B cells, regulatory cells (Tregs), Th17 cells, central memory T cell (Tcm), dendritic cells (DC), and neutrophils were negatively correlated with the expression of RPL19. While NK CD56bright cells were positively correlated with the expression of RPL19 (Figures 7A–F). To further assess the clinical impact of RPL19, the effect of immune infiltration on survival was analyzed (Supplementary Figures 5, 6). Most immune cells were protective factors except T helper cells and Th2 cells (Figure 7G). And the immune infiltration was significantly suppressed in HCC with high expression of RPL19.

## Discussion

Because of the role of genetic factors in the occurrence, development, progression and prognosis of HCC, we can study the functions of genes at the whole genome level through microarrays and high-throughput sequencing (Byron et al., 2016). As a systematic biological method used to describe how clinical features are related to genes, in this study, WGCNA was used to study gene co-expression in HCC and normal tissues. WGCNA divided genes into multiple modules by analyzing the relationship between genes. Then, correlation analysis between these modules and different phenotypes was used to determine the molecular characteristics of the specific phenotype. To date, WGCNA has been used to explore hub genes and tumor biomarkers of many cancers, such as bladder cancer (Jiang et al., 2020), prostate cancer (Wei et al., 2020), oral squamous cell carcinoma (Dai et al., 2020) and pancreatic cancer (Wang et al., 2020). In regard to HCC, many studies have been conducted to identify the hub genes correlated with its progression and prognosis. Gu et al. performed WGCNA based on the TCGA database and found two hub modules (turquoise module and blue module) and 13 hub genes (SNRPD2, PRR11, SKA3, etc.) that have a high correlation with progression and prognosis in HCC (Gu et al., 2020). They chose the GEO dataset GSE6764 to validate these genes. Moreover, they conducted real-time PCR to figure out the expression difference in HCC and paracancerous tissues. Li et al. (2020) performed WGCNA based on the GEO dataset GSE54238 and screened four hub genes (TDRKH, TARBP1, STK39, and SOX4) that were correlated with immune infiltration and found that these four genes had certain diagnostic value for HCC. The TCGA database was chosen to validate these genes. Previous studies have shown that 10 genes (CD8A, GMPS, STAT3, ERBB2, ACACA, ALB, EGFR, TGFB1, KRAS, and BCL2) are involved in multiple pathways, including cell adhesion, migration, locomotion, and differentiation, in the occurrence and progression of HCC (Zhang et al., 2017). Research on multiple different databases help us to understand the mechanism of the occurrence and development of HCC more comprehensively and guide clinical treatment.

In this study, we extracted co-expression networks of groups of genes from GSE39791 to conduct WGCNA and obtained 17 co-expression modules. Through further analysis, we found that three modules (green, blue, and purple) were most relevant to cancer samples. The green and blue modules were involved in multiple cancer-related KEGG pathways. We ultimately identified four genes, RPL19, RPS12, RPL35A, and RPL27A, and all four genes are related to ribosomes and are highly expressed in HCC tissues. The high expression of the four genes was related to the poor prognosis of patients. In addition, we identified the enrichment pathways based on the high expression of RPL19.

The four hub genes we discovered have been studied in multiple tumors. RPL19, which is a tumor-specific antigen of lung adenocarcinoma (Kuroda et al., 2010), can also be used as a prognostic biomarker for prostate cancer (Bee et al., 2006), colorectal cancer (Huang et al., 2008), and diffuse large B-cell lymphoma (Yan et al., 2019). One study (Bee et al., 2006) proposed that the expression of RPL19 in malignant prostate cancer cells was significantly higher than that in prostate cells. The degree of RPL19 staining in cancer tissues was significantly higher than that in normal prostate tissues and benign prostatic hyperplasia tissues, and the survival time of patients with high RPL19 expression was shortened, suggesting that RPL19 could be used as a biomarker for the diagnosis and prognosis of prostate cancer. Another study showed that the expression of cytokeratin 19 (CK19) and RPL19 in the stool of patients with advanced colorectal cancer was significantly increased. The simultaneous detection of two markers could better identify high-risk populations who are prone to metastasis (Huang et al., 2008). It was also found in lung cancer that the level of RPL19 mRNA expression in normal lung tissues was lower than that in lung cancer tissues (Kuroda et al., 2010), and the overexpression RPL19 was positively correlated with interferon IFN-γ. The synthesis of cyclin D1 and D3 decreased after RPL19 expression was inhibited. Therefore, the decrease in the proliferation of lung cancer cell lines caused by RPL19 knockdown may occur through inhibition of the cell cycle (Kuroda et al., 2010). We conducted further studies on RPL19 and obtained consistent results with the bioinformatics analysis on the TMA. RPL27A is a tumor biomarker for colorectal cancer (Yajima et al., 2007). High expression of the RPL27A gene will increase the risk of colorectal cancer (Takemasa et al., 2012). RPL27A was also identified as a biomarker for squamous cervical cancer (Fjeldbo et al., 2016). The RPL35A gene is located at chromosome 3q29-qter (Colombo et al., 1996), and almost all studies have suggested that Diamond-Blackfan anemia is caused by deletion of the RPL35A gene (Farrar et al., 2008, 2011; Gianferante et al., 2020). Finally, the RPS12 gene has been shown to be related to the biological functions of various plants (Lee et al., 2019) and insects (Ji et al., 2019; Kirby and Koslowsky, 2020). Studies have shown that RPS12 gene deletion is associated with diffuse large B-cell lymphoma (Derenzini et al., 2019). The RPS12 gene has been demonstrated to be a hypoxia-related gene, and high expression of the RPS12 gene increases the risk of gastric cancer (Chen et al., 2013), squamous cell carcinoma (Fjeldbo et al., 2016) and HCC (Wang et al., 2009).

Ribosomal proteins are the main component of ribosomes and play an important role in protein biosynthesis in cells. Ribosomes participate in DNA repair, cell development regulation and cell differentiation (Petibon et al., 2021). In addition, the dysregulation of RPs affects the progression and prognosis of multiple diseases (Bolze et al., 2013; Ebright et al., 2020). Moreover, there are many studies on the relationship between ribosomal proteins and HCC. Researchers analyzed HCC cell lines and tissue samples and found that the expression levels of RPS3A in HCC cell lines and tissues were higher than those in normal liver cells and adjacent tumor-free tissues, and patients with high RPS3A expression had shorter OS and RFS than patients with low RPS3A expression (Zhou et al., 2020c). Guo et al. (2018) proposed that ribosomal protein S15a promotes tumor angiogenesis by enhancing Wnt/β-catenin-induced FGF18 expression in HCC. It has been reported that RPS11 is highly expressed in liver cancer tissues, and its high expression indicates a poor prognosis (Zhou et al., 2020b). On the other hand, studies (Chen et al., 2020; Zhou et al., 2020a) have shown that ribosomal proteins can be used as intermediate targets to inhibit the progression of HCC. Therefore, in the future, ribosomal proteins may become important targets in the diagnosis, treatment and prognosis of HCC.

Through the functional annotation and enrichment pathway analysis of RPL19, we found that high RPL19 expression suppressed bile acid metabolism and activated the cell cycle. Bile acids are produced in the liver and metabolized by enzymes derived from gut bacteria. They are essential for maintaining healthy gut microbiota, balancing lipid and carbohydrate metabolism, insulin sensitivity and innate immunity (Li et al., 2017). Increasing evidence has shown that bile acids play a vital role in the occurrence and progression of HCC (Jia et al., 2018). Studies have shown that the inhibition of bile acid metabolism can lead to cholestasis and increase the risk of HCC (Knisely et al., 2006). On the other hand, ursodeoxycholic acid can prevent liver cholestasis, thereby exerting its hepatoprotective effect (Beuers et al., 2015). Bioinformatic analysis indicated that high DDX11 expression was closely related to the G2-M phase transition of the cell cycle and DNA replication. Uncontrolled excessive proliferation is one of the main characteristics of tumor cells. Multiple studies have shown that the cell cycle pathway of liver cancer cells is significantly enhanced (Rebouissou and Nault, 2020), and the progression of liver cancer can be inhibited by inhibiting the cell cycle (Lee et al., 2018). Moreover, the immune infiltration analysis showed that the immune infiltration was significantly suppressed in HCC with high expression of RPL19. Ma et al. (2020) found that the expression of Aurora kinase A and ninein-interacting protein (AUNIP) was positively correlated with the degree of infiltration of dendritic cells, macrophages, neutrophils, CD8 + T cells, CD4 + T cells and B cells in HCC. Subsequent study showed that TANK-binding kinase 1 (TBK1) was a potential target for HCC by enhancing tumor immune infiltration (Jiang et al., 2021).

We acknowledge that there were some limitations and shortcomings to this study. First, WGCNA is based on highly correlated key modules to conduct the analysis, some key genes with low correlation may be missed. In addition, in this study we chose three hub modules with the highest correlation coefficient and positive correlation for model construction. In the process, we missed some highly negatively correlated modules (such as turquoise model in Figure 3). In the future research, we need to pay attention to the genes in these modules. Second, in this study, we identified four hub genes through WGCNA. We comprehensively considered the expression level, protein level, and gene function of the four genes. Only RPL19 has been validated in this study, the functions of the other three molecules (RPL35A, RPL27A, and RPS12) need further studies. Third, we only analyzed the relationship between the expression and clinical features but did not verify these findings through in vivo and in vitro experiments. Finally, we only explored the underlying mechanism based on bioinformatic prediction. The molecular mechanism of up-regulated RPL19 promoting the progression of HCC remains a subject for further study.

## Conclusion

In conclusion, WGCNA was used to construct a co-expression gene network and revealed four hub genes (RPL19, RPL35A, RPL27A, and RPS12) that were highly expressed in HCC and whose expression were negatively correlated with HCC prognosis. Then, the effect of high RPL19 expression on the prognosis of HCC was verified through a TMA. Enrichment analysis revealed that cell cycle pathways were significantly enriched, and bile acid metabolism-related pathways were significantly down-regulated when RPL19 was highly expressed. The immune infiltration analysis showed that the immune infiltration was significantly suppressed in HCC with high expression of RPL19. As a result, RPL19 may be a molecular biomarker and drug target for the early diagnosis and prognosis of HCC. However, the mechanism by which RPL19 promotes the occurrence and development of HCC through the above pathways is still unknown, which is our next key research direction.

## Data Availability Statement

The datasets presented in this study can be found in online repositories. The names of the repository/repositories and accession number(s) can be found in the article/Supplementary Material.

## Ethics Statement

The studies involving human participants were reviewed and approved by Institutional Review Board of The First Affiliated Hospital of Zhengzhou University. The patients/participants provided their written informed consent to participate in this study.

## Author Contributions

ZY and ZR designed the study. BR, JL, and TR provided equal contributions to the research design, data analysis, and article writing. JY, GZ, LL, HW, and MH revised the manuscript. All authors contributed to the article and approved the submitted version.

## Conflict of Interest

The authors declare that the research was conducted in the absence of any commercial or financial relationships that could be construed as a potential conflict of interest.

## Footnotes

1 http://www.ncbi.nlm.nih.gov/geo/

2 https://tcga-data.nci.nih.gov/tcga/

3 http://cbdm.mdc-berlin.de/tools/hippie/hippie_current.txt

4 http://metascape.org

5 https://www.kegg.jp/

6 http://geneontology.org/

7 http://www.gsea-msigdb.org/gsea/msigdb/genesets.jsp?collection=H

8 www.r-project.org

## Supplementary Material

Supplementary Figure 1 The 11 modules were enriched in 121 KEGG pathways. KEGG, Kyoto Encyclopedia of Genes and Genomes.

Supplementary Figure 2 Distribution of the co-expression network degree.

Supplementary Figure 3 Expression levels of the four hub genes in different stages of liver disease. **(A)** Expression level of RPL19 in different stages of liver diseases. **(B)** Expression level of RPL27A in different stages of liver diseases. **(C)** Expression level of RPL35A in different stages of liver diseases. **(D)** Expression level of RPS12 in different stages of liver diseases. ^∗^*P* < 0.05, ^∗∗^*P* < 0.01.

Supplementary Figure 4 Relationship between the expression levels of the four hub genes and the prognosis of HCC patients with different TNM stages. **(A)** Relationship between RPL19 expression and the OS and RFS of HCC patients according to TNM stage (stages I∼II and III∼IV). **(B)** Relationship between RPL27A expression and the OS and RFS of HCC patients according to TNM stage (stages I∼II and III∼IV). **(C)** Relationship between RPL35A expression and the OS and RFS of HCC patients according to TNM stage (stages I∼II and III∼IV). **(D)** Relationship between RPS12 expression and the OS and RFS of HCC patients according to TNM stage (stages I∼II and III∼IV). OS, overall survival. RFS, relapse- free survival.

Supplementary Figure 5 Overall survival curves of the immune infiltrating cells in HCC. Kaplan-Meier analysis showing the correlation between the immune cells scores and the OS of HCC patients. OS, overall survival.

Supplementary Figure 6 Progression free survival curves of the immune infiltrating cells in HCC. Kaplan-Meier analysis showing the correlation between the immune cells scores and the PFS of HCC patients. PFS, progress free survival.

Supplementary Table 1 All 31,334 genes identified.

Supplementary Table 2 All 7,814 DEGs.

Supplementary Table 3 Statistical results of genes corresponding to each module.

Supplementary Table 4 Distribution of 7,814 selected genes in the different modules.

Supplementary Table 5 Eleven modules were enriched in 121 KEGG pathways.

Supplementary Table 6 All genes whose co-expression values were greater than 0.1.

Supplementary Table 7 Eleven modules were enriched in 121 KEGG pathways.

Supplementary Table 8 Human PPI data from the HIPPIE database.

Supplementary Table 9 Human PPI network.
