# Supplementary figures and images for: RPL19 Is a Prognostic Biomarker and Promotes Tumor Progression in Hepatocellular Carcinoma
Source: Front Cell Dev Biol. 2021 Jul 19;9:686547. doi: 10.3389/fcell.2021.686547 (PMC8327752; doi:10.3389/fcell.2021.686547)

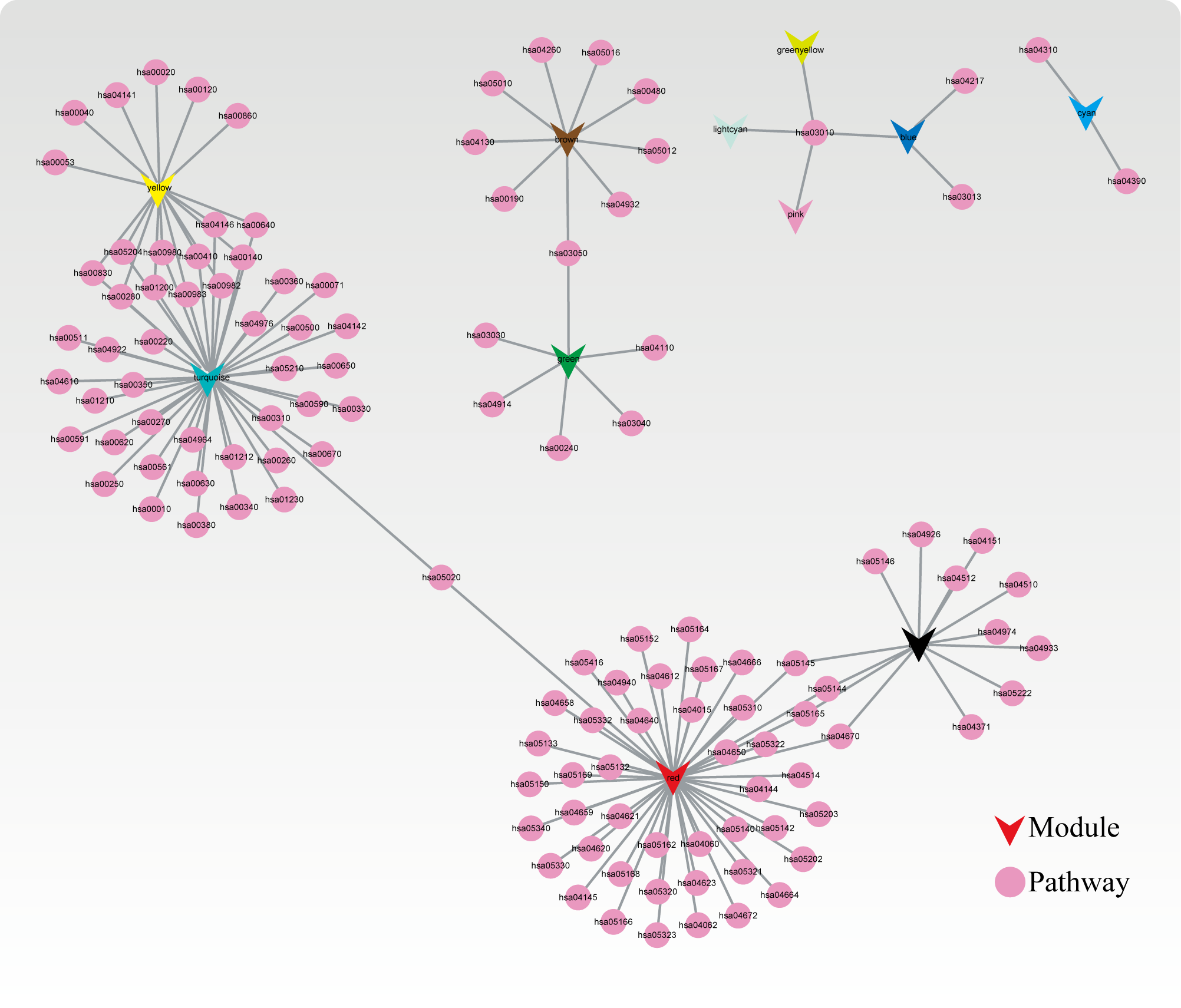

Supplement: Supplementary Figure 1 — The 11 modules were enriched in 121 KEGG pathways. KEGG, Kyoto Encyclopedia of Genes and Genomes. [file Image_1.TIF]

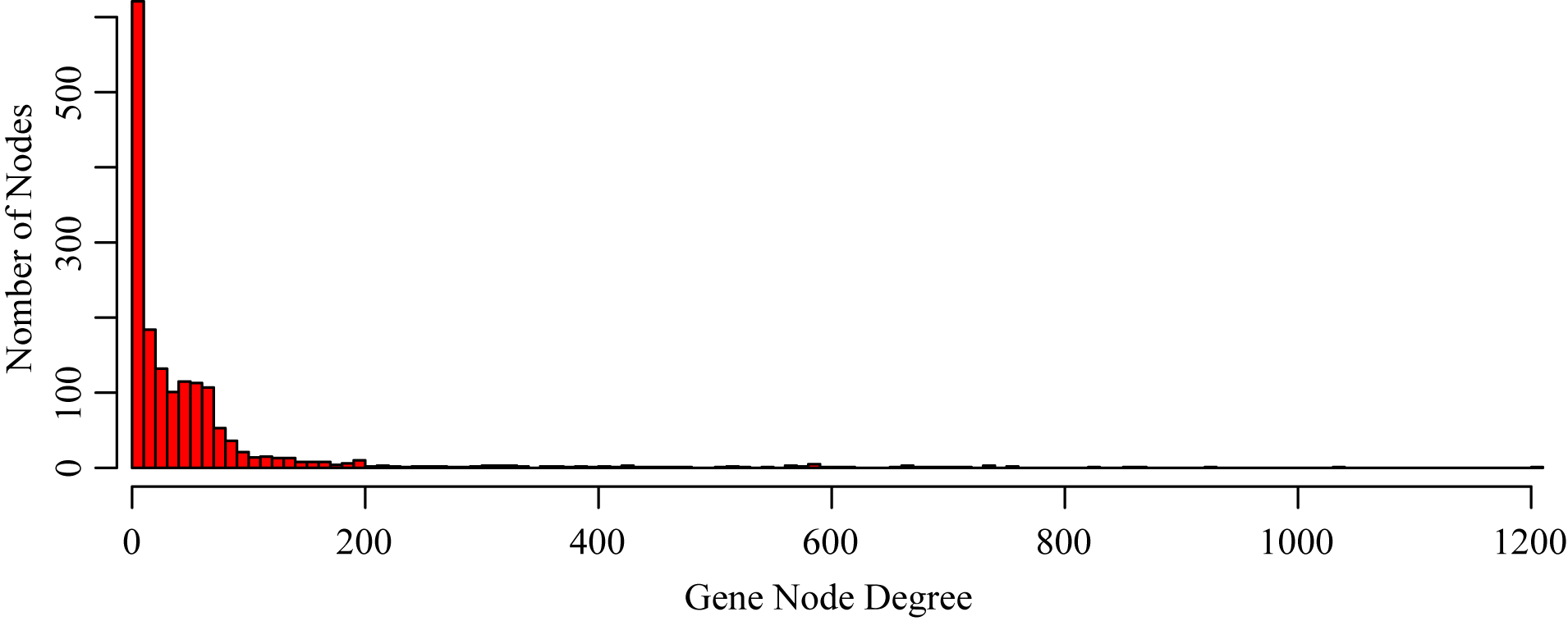

Supplement: Supplementary Figure 2 — Distribution of the co-expression network degree. [file Image_2.TIF]

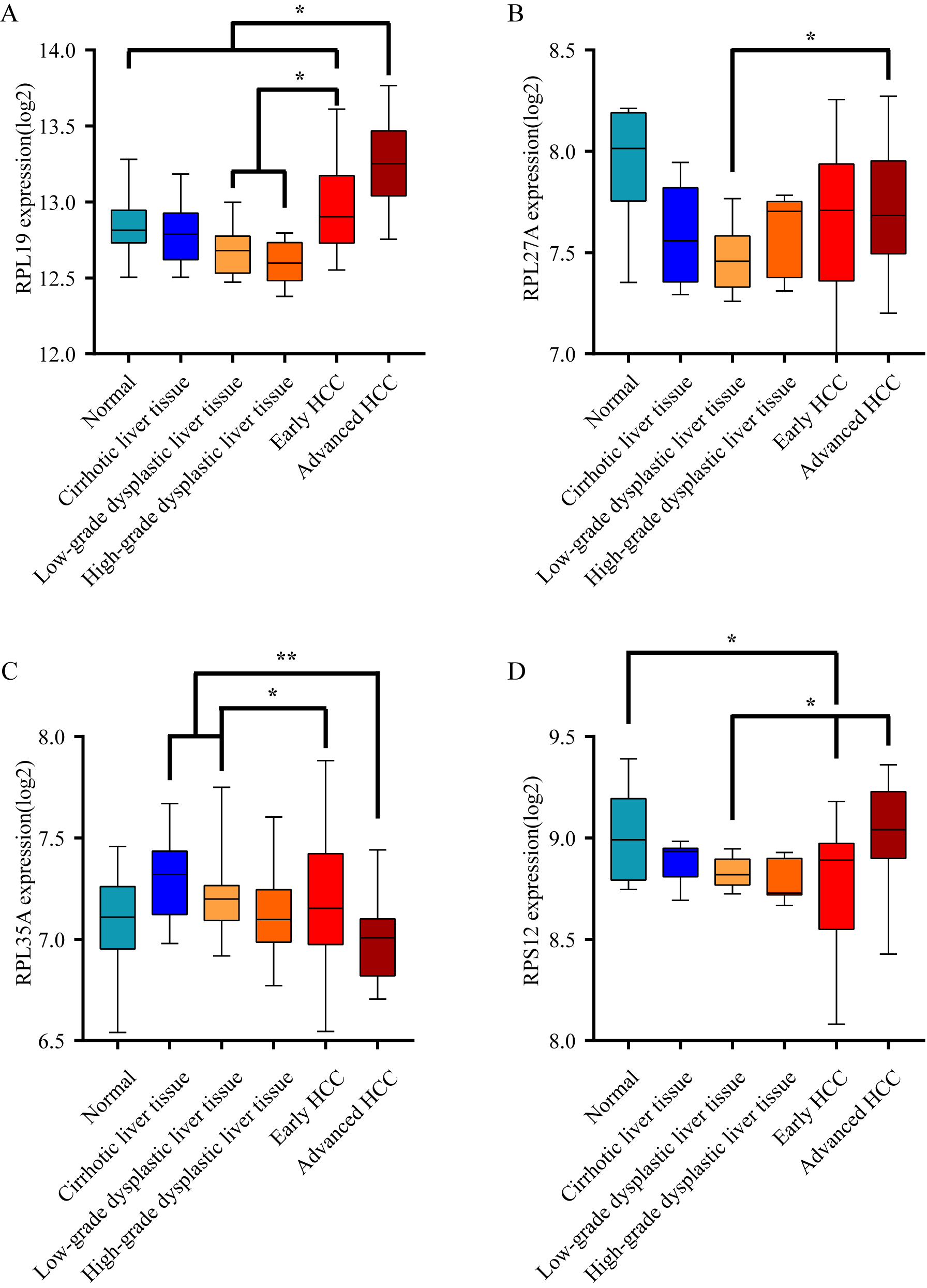

Supplement: Supplementary Figure 3 — Expression levels of the four hub genes in different stages of liver disease. (A) Expression level of RPL19 in different stages of liver diseases. (B) Expression level of RPL27A in different stages of liver diseases. (C) Expression level of RPL35A in different stages of liver diseases. (D) Expression level of RPS12 in different stages of liver diseases. ∗P < 0.05, ∗∗P < 0.01. [file Image_3.TIF]

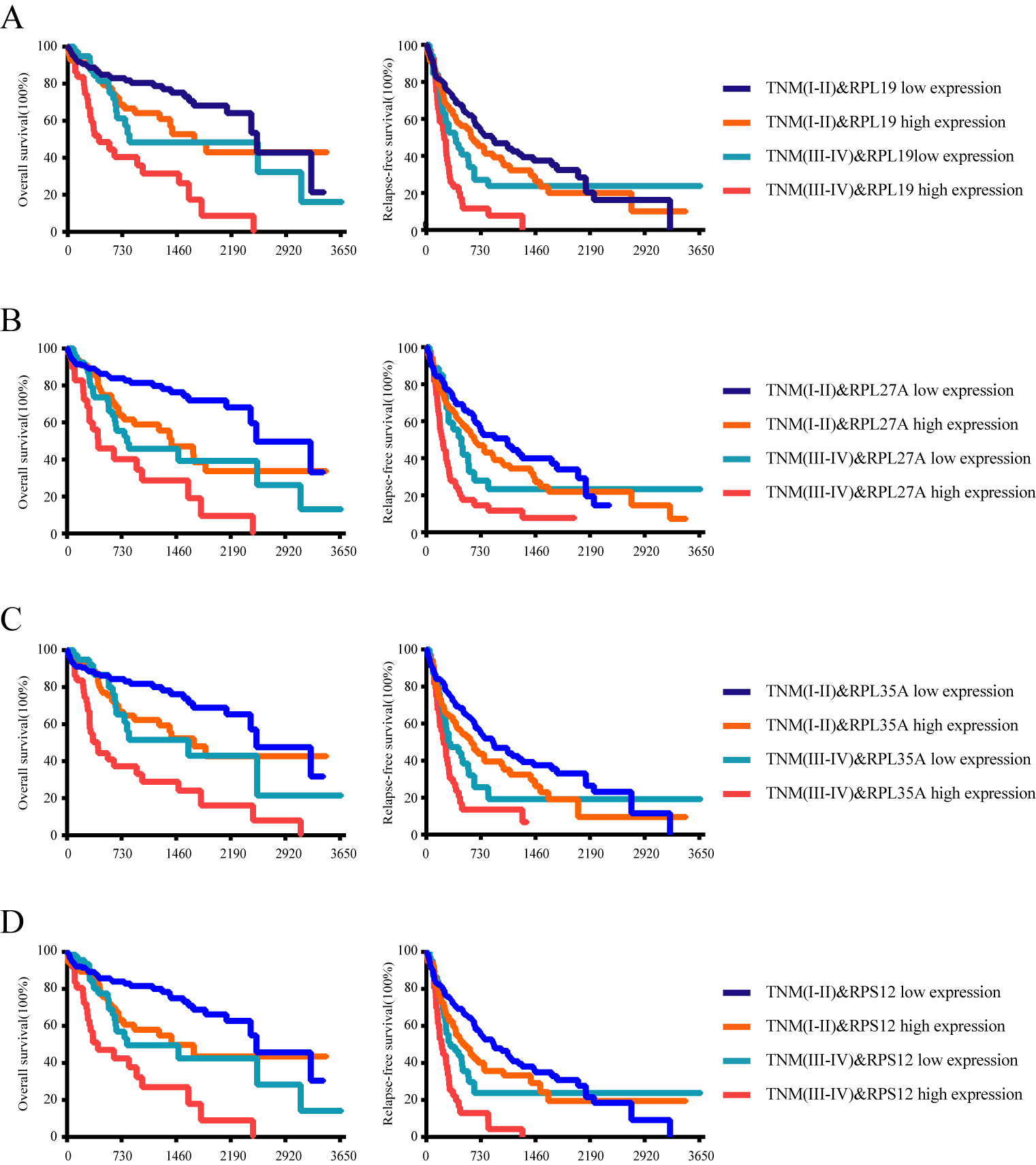

Supplement: Supplementary Figure 4 — Relationship between the expression levels of the four hub genes and the prognosis of HCC patients with different TNM stages. (A) Relationship between RPL19 expression and the OS and RFS of HCC patients according to TNM stage (stages I∼II and III∼IV). (B) Relationship between RPL27A expression and the OS and RFS of HCC patients according to TNM stage (stages I∼II and III∼IV). (C) Relationship between RPL35A expression and the OS and RFS of HCC patients according to TNM stage (stages I∼II and III∼IV). (D) Relationship between RPS12 expression and the OS and RFS of HCC patients according to TNM stage (stages I∼II and III∼IV). OS, overall survival. RFS, relapse- free survival. [file Image_4.TIF]

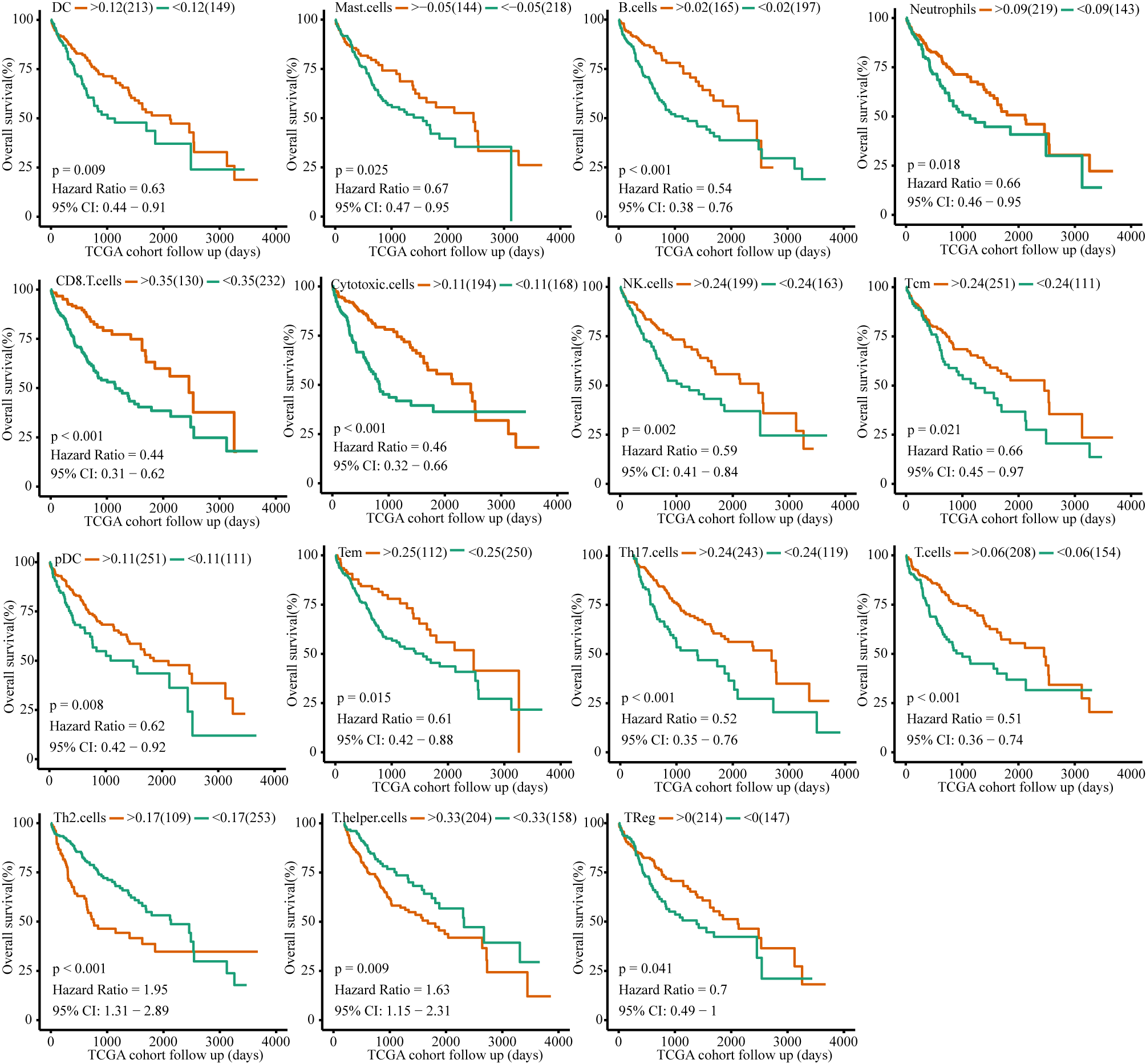

Supplement: Supplementary Figure 5 — Overall survival curves of the immune infiltrating cells in HCC. Kaplan-Meier analysis showing the correlation between the immune cells scores and the OS of HCC patients. OS, overall survival. [file Image_5.TIF]

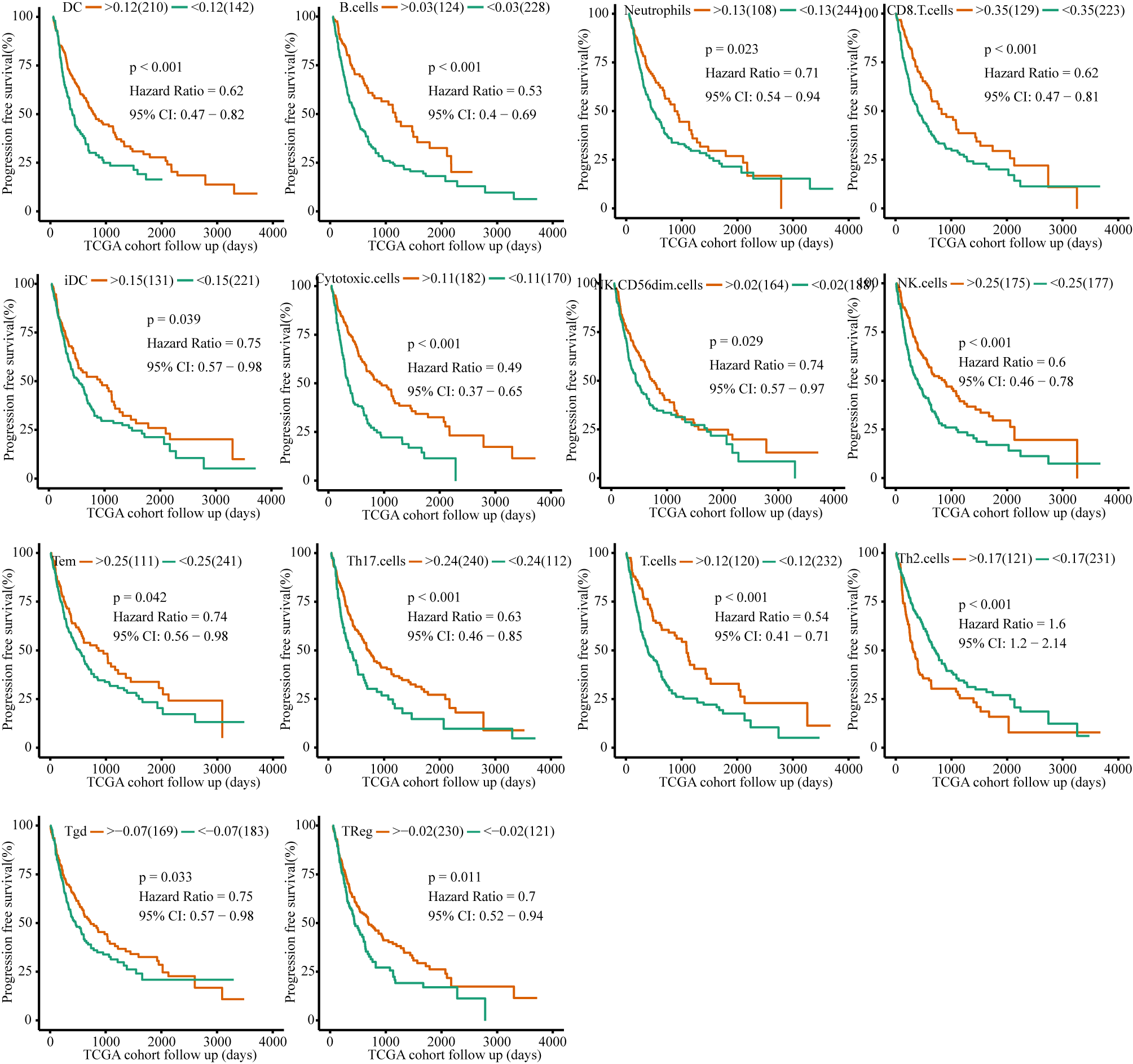

Supplement: Supplementary Figure 6 — Progression free survival curves of the immune infiltrating cells in HCC. Kaplan-Meier analysis showing the correlation between the immune cells scores and the PFS of HCC patients. PFS, progress free survival. [file Image_6.TIF]
